# Supplementary material for: Temporal loss boundary engineered photonic cavity
Source: Nat Commun. 2021 Nov 26;12:6940. doi: 10.1038/s41467-021-27014-z (PMC8626434; doi:10.1038/s41467-021-27014-z)
Supplement: Supplementary file 1 — Supplementary Information [file 41467_2021_27014_MOESM1_ESM.pdf]

Supplementary materials for temporal loss boundary engineered photonic cavity

Longqing Cong[1,*], Jiaguang Han[2], Weili Zhang[3], and Ranjan Singh[4]

[1]*Department of Electrical and Electronic Engineering, Southern University of Science and Technology, Shenzhen 518055, China*

[2]*Center for Terahertz Waves and College of Precision Instrument and Optoelectronics Engineering, Tianjin University, and the Key Laboratory of Optoelectronics Information and Technology (Ministry of Education), Tianjin 300072, China*

[3]*School of Electrical and Computer Engineering, Oklahoma State University, Stillwater, OK 74078, USA*

[4]*Division of Physics and Applied Physics, School of Physical and Mathematical Sciences and Centre for Disruptive Photonic Technologies, The Photonics Institute, Nanyang Technological University, Singapore 637371, Singapore*

[*]Email: conglq@sustech.edu.cn

1. Lorentzian fitting to the spectra in regime I and regime II

The unperturbed TE and TM modes of the metamaterial cavity are described by electric and magnetic dipole polarizations that can be numerically modelled by Lorentzian lineshape. The transmission spectrum of the coupled modes is analytically expressed as a function of angular frequency ω as[1]:

$$t = 1 + \frac{2i\cdot\gamma_r^e\cdot\omega}{\left(\omega_0^e\right)^2-\omega^2-2i(\gamma_r^e+\gamma_n^e)\omega} + \frac{2i\cdot\gamma_r^m\cdot\omega}{\left(\omega_0^m\right)^2-\omega^2-2i(\gamma_r^m+\gamma_n^m)\omega}, \tag{S1}$$

where γ_r, γ_n are loss rates of radiative and nonradiative losses, respectively. TE and TM modes are written as two Lorentzian dipoles (superscripts *e* and *m* for TE and TM modes, respectively) with central frequencies at ω_0^e, ω_0^m. By using the model of Eq. S1, we fitted the transmission spectra of metamaterial cavity measured in regime II and regime I as shown in supplementary Fig. 1a and 1b, respectively. We observe the clear discrepancy between the fitting lineshape and measured spectrum in regime II indicating that the mode profile is no longer a Lorentzian, but distorted by the sudden merging of temporal boundary.

After perturbation, the cavity mode becomes a mixture of harmonic dipole and the perturbation function. Therefore, the resultant cavity possesses an anharmonic oscillation that will involve high-order frequency components. On the other hand, the spectrum in regime I under the pump fluence of 0.63 μJ·cm^{-2} reveals a much better agreement with the fitting Lorentzian lineshape, which means both two modes are modulated without distorting the mode profile.

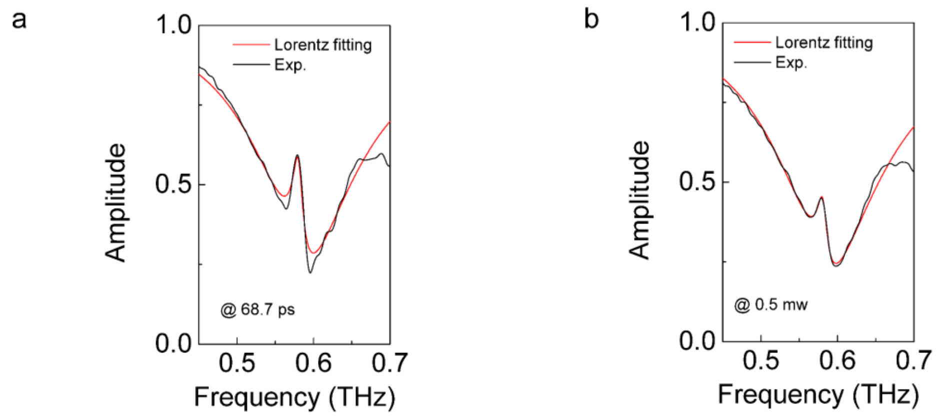

Supplementary Fig. 1. Numerical fitting by coupled Lorentzian dipole model. a. Coupled Lorentzian lineshape fitting and transmission spectrum of cavity that is modulated by the temporal boundary at t_r = 68.7 ps in regime II; **b.** Lorentzian lineshape fitting curve and transmission spectrum of cavity that is modulated in regime I under a pump fluence of 0.63 μJ·cm^{-2}.

2. Transition from coupled to decoupled cavity in regime I

It is straightforward to understand the decoupling cavity in regime II where the higher *Q* mode is completely quenched and the lower *Q* mode sustains with negligible perturbation from the temporal boundary. While in regime I both two modes are damped synchronously under the pump, the cavity can still be decoupled due to the distinct damping thresholds of pump fluence for the two modes of different *Q*s. Higher (lower) *Q* mode shows more (less) sensitive to damping and thus will be completely quenched at a relative lower (higher) pump power. Therefore, the two-mode cavity can be decoupled in regime I by increasing the pump fluence to the threshold of TE mode in order to quench it, and leaving the low *Q* mode (TM) sustaining. We probed the threshold to decouple the two modes in regime I

and the measured transmission spectra. It is shown that the high Q TE mode quenches at a pump power of 4 mW (5 μJ·cm^{-2}) and the TM dipole sustains in the decoupled cavity. Similarly, the accompanied phase spectra exhibit the evolution of cavity from coupled to decoupled state indicated by the phase coverage transition from 360° to 180°.[2]

The distinction between regime I and regime II can also be directly observed from the temporal profiles. In regime II (supplementary Fig. 2a), there is no doubt that the main pulses containing most spectral energy are unperturbed as long as the temporal barrier is injected after them. Only the long-lasting oscillations afterwards for the high Q mode will be disturbed by the temporal barrier, and therefore the temporal spectrum is in fact a superposition of cavity modes and external perturbation. However, the pulse will experience a uniform modulation in the entire lifetime in regime I, and thus all the spectral components are modulated uniformly as indicated by supplementary Fig. 2b, sustaining their Lorentzian lineshapes.

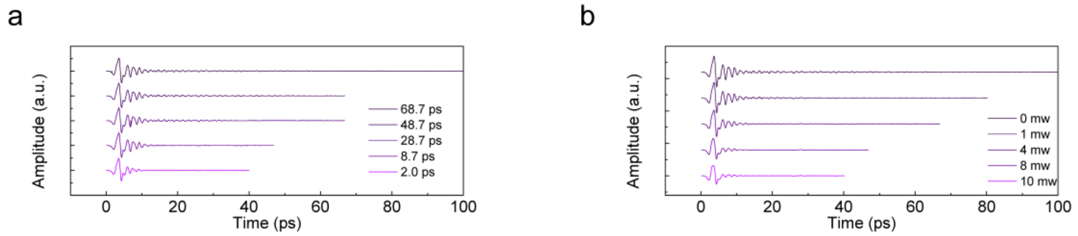

Supplementary Fig. 2. Directly observe the modulation of temporal profiles in regime II and regime I. a. Temporal profiles in regime II by injecting temporal barriers at different timings after which the oscillations are accelerated to damp out. **b.** Temporal profiles in regime I by increasing the pump power from 0 mw to 10 mw (12.5 μJ·cm^{-2}). The entire temporal profiles are uniformly damped with the strength depending on the pump power.

3. Frequency ripples of cavity due to Heaviside function

Here we show that the ripples in frequency spectra in regime II is majorly contributed by the perturbation of nonequilibrium photocarriers. In our experiments, the accumulation period of photocarriers is 14 ps that cannot be ignored, and so we numerically expressed the dynamics as an exponential function after t_r as shown in supplementary Fig. 3a. Before

t_r the Heaviside function is equal to 1 which has no perturbation on the cavity. Here, the function described in supplementary Fig. 3a represents the evolution of cavity dynamics coupling to the original temporal profile of cavity modes. Then the measured transmission spectra in regime II can be modelled as a simple superposition of unperturbed temporal profile and the Heaviside function, and the FFT of which results in the perturbed frequency spectrum with ripples (supplementary Fig. 3b). To further verify the argument, we show the FFT spectra of the Heaviside function in the interested frequency band as shown in supplementary Fig. 3b. We observe the periodic ripples whose period depends on the injection timing of temporal barrier t_r coincidence to the evolution of perturbed spectra in experiments.

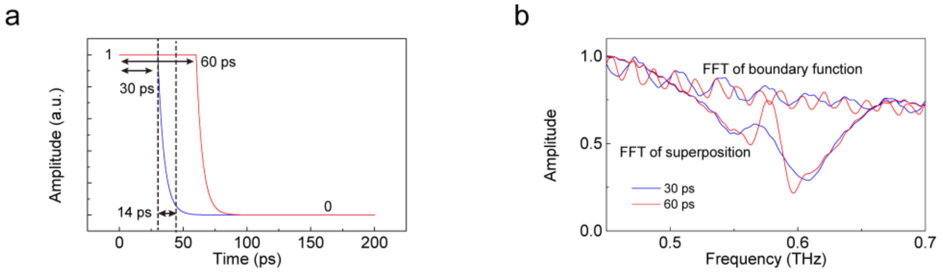

Supplementary Fig. 3. Interpretation of ripples in the perturbed transmission spectra in regime II. a. Numerical representation of temporal barrier profiles with timing at t_r = 30 ps and t_r = 60 ps. The cavity dynamics after t_r is expressed as an exponential function that decreases from 1 to 0 within 14 ps. **b.** Fourier transforms of the temporal boundaries reveal the oscillations in frequency domain whose period is determined by injection timing t_r. The coupling of temporal boundary and cavity photons is interpreted as a convolution function between the boundary function and spectrum of unperturbed cavity.

4. Symmetric pattern without P_2 component

As mentioned above, the coupling of temporal boundary to the cavity leads to the decoration of the perturbed spectrum, and the period of ripples depends on the injection timing. Here in supplementary Fig. 4a, we present the full evolution of the perturbed spectra (transmission difference relative to the unperturbed spectrum) by continuously adjusting the delay time. One feature should be noted that the spectral pattern reveals mirror

symmetry relative to the central frequency of the dipole. In the experiments, however, the ripples reveal asymmetry in the frequency spectra (Fig. 3a) with more pronounced ripples at higher frequencies. This should be contributed to the newly generated dipole polarization P_2 at higher frequency due to the sudden decrease of cavity refractive index, and therefore it is necessary to apply the two-dipole equations to model the cavity dynamics.

5. Cavity *Q* on perturbation strength

The Q of the cavity mode plays a key role in the determining the perturbation strength of temporal boundary on the perturbed cavity spectrum. The contrast is shown in supplementary Fig. 4b and 4c by calculating the modulation of two cavity modes with the same temporal boundary, where the $Q = 60$ cavity mode reveals a much slighter modulation than that of $Q = 120$ one with the same injection timing.

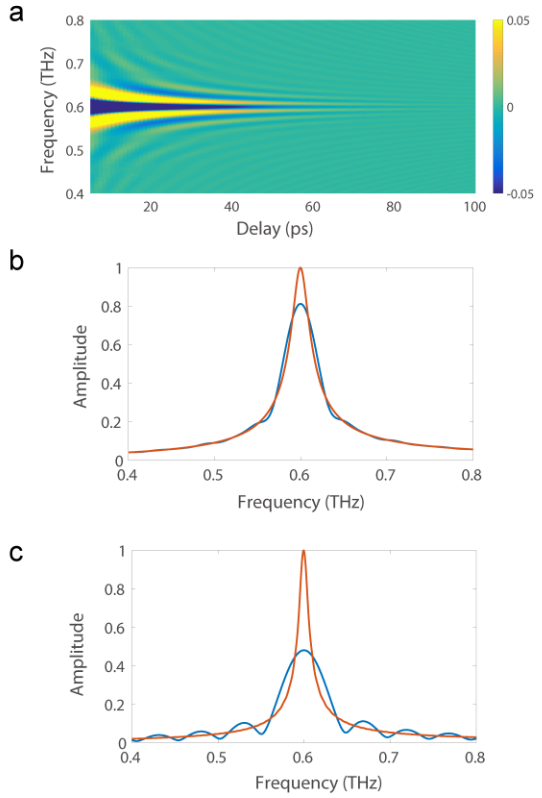

Supplementary Fig. 4. Interpretation of P_2 component and cavity mode Q on the spectral features. a. The calculated transmission difference spectra of a single dipole cavity at different

temporal barrier delays. Only P_1 part is considered here, and we observe a symmetric pattern of the ripple induced by the temporal boundary. **b. and c.** Comparison of the modulation between two dipoles with different Qs at t_r = 20 ps. The higher Q dipole reveals a much more profound modulation.

6. Carrier induced absorption loss and index changing

The ansatz of the generation of a new dipole component is due to the fact that refractive index of silicon is modified by the temporal perturbation which thus tunes the cavity frequency in analog to changing the tone of guitar by adjusting string length after plucked. The refractive index change comes from two main mechanisms: Drude and band filling terms due to injection of free carriers. The pump fluence in our experiments is set in a low level with a low density of free carriers, and we thus ignore the effect of band filling and only consider the Drude term. The injection of free carriers will induce the decrease of refractive index and increase of absorption according to the Drude term:

$$\Delta n = -\frac{e^2}{2\omega^2 \varepsilon_0 n}\left(\frac{\Delta N_e}{m_e^*} + \frac{\Delta N_h}{m_h^*}\right), \tag{S2}$$

$$\Delta \alpha = \frac{e^3}{\omega^2 c \varepsilon_0 n}\left(\frac{\Delta N_e}{m_e^{*2}\mu_e} + \frac{\Delta N_h}{m_h^{*2}\mu_h}\right), \tag{S3}$$

where subscripts e and h refer to electron and hole, respectively, and ΔN is the change of density of electrons (holes); m^* is the effective mass of electrons (holes); μ is the mobility of electrons (holes); n is steady refractive index of silicon; c is the vacuum speed of light; ε_0 is the permittivity of free space; ω is the angular frequency. Since the sample was fabricate with intrinsic silicon, electrons and holes should be equal $\Delta N_e = \Delta N_h = N/2$. Using the numerical fitting to experimental data, the P_2 component has a central frequency of 0.590 THz that is shifted by 0.009 THz from unperturbed dipole frequency at 0.581 THz, therefore, we estimated the change of effective refractive index as $\Delta n = n\Delta\omega/\omega$, i.e., $\Delta n \approx$ -0.058. The carrier density was then estimated to be ~ 1×10^{14} cm^{-3}.[3, 4] Note that the pump energy can only be absorbed on the shallow surface of the silicon cylinder resonators (with

an estimated depth ~ 10 μm), and the estimation of refractive index change is an effective one which would not be uniformly distributed in the entire silicon resonators, and so does the carrier density.

7. Direct observation of perturbation induced changes

Here we show the direct observation of the temporal boundary induced modulation of time-domain profiles ($\Delta E = E_0 - E_p$ where E_0 is the EO sampling electric field in steady state and E_p is the electric field at various temporal boundary delays) at different delay times t_r . It is manifested that the signals are not perturbed before the injection of temporal boundary as divided by the dashed line, while the perturbed region reveals oscillations with frequency of the TE mode. This is verified after performing Fourier transform of the difference signals as shown in supplementary Fig. 5b, and the modulation strength depends on the delay times.

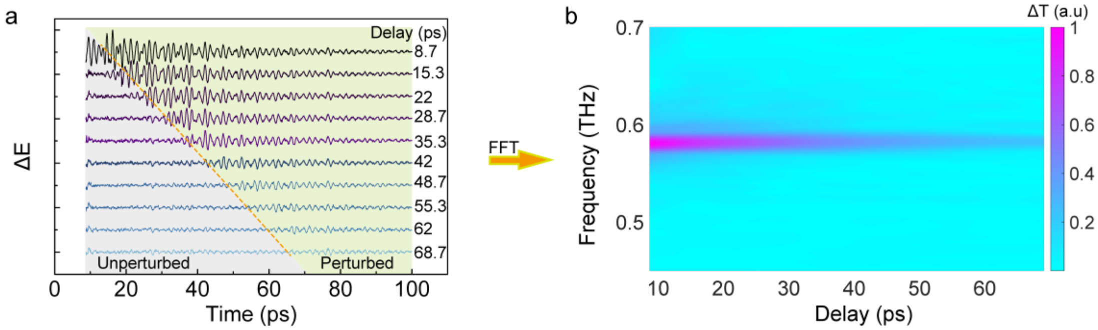

Supplementary Fig. 5. Perturbation induced change in time domain. a. Output electric field difference induced by temporal boundary (perturbation) at various delay times ($\Delta E = E_0 - E_p$). **b.** Fourier transform of ΔE at various delay times showing the gradual modulation of TE mode.

Reference

1. Decker M., *et al.* High-Efficiency Dielectric Huygens' Surfaces. *Adv. Opt. Mater.* **3**, 813-820 (2015).
2. Rahimzadegan A., *et al.* Disorder-Induced Phase Transitions in the Transmission of Dielectric Metasurfaces. *Phys. Rev. Lett.* **122**, 015702 (2019).
3. Soref R., Bennett B.. Electrooptical effects in silicon. *IEEE J. Quantum Electron.* **23**, 123-129 (1987).

4. Shcherbakov M.R., *et al*. Ultrafast all-optical tuning of direct-gap semiconductor metasurfaces. *Nat. Commun.* **8**, 17 (2017).
